# Enhanced glioblastoma immunotherapy via SMAC mimetic dose escalation and TGFβ blockade

**DOI:** 10.1093/noajnl/vdaf253

**Published:** 2025-12-10

**Authors:** Kyle Malone, Melanie Dugas, Nathalie Earl, Tommy Alain, Robert G Korneluk, Eric LaCasse, Shawn T Beug

**Affiliations:** Apoptosis Research Centre, Children’s Hospital of Eastern Ontario Research Institute, Ottawa, Ontario K1H 8L1, Canada; Department of Biochemistry, Microbiology and Immunology, University of Ottawa, Ottawa, Ontario K1H 8M5, Canada; Centre for Infection, Immunity and Inflammation, University of Ottawa, Ottawa, Ontario K1H 8M5, Canada; Ottawa Institute of Systems Biology University of Ottawa, Ottawa, Ontario K1H 8M5, Canada; Apoptosis Research Centre, Children’s Hospital of Eastern Ontario Research Institute, Ottawa, Ontario K1H 8L1, Canada; Department of Biochemistry, Microbiology and Immunology, University of Ottawa, Ottawa, Ontario K1H 8M5, Canada; Centre for Infection, Immunity and Inflammation, University of Ottawa, Ottawa, Ontario K1H 8M5, Canada; Apoptosis Research Centre, Children’s Hospital of Eastern Ontario Research Institute, Ottawa, Ontario K1H 8L1, Canada; Department of Biochemistry, Microbiology and Immunology, University of Ottawa, Ottawa, Ontario K1H 8M5, Canada; Centre for Infection, Immunity and Inflammation, University of Ottawa, Ottawa, Ontario K1H 8M5, Canada; Ottawa Institute of Systems Biology University of Ottawa, Ottawa, Ontario K1H 8M5, Canada; Apoptosis Research Centre, Children’s Hospital of Eastern Ontario Research Institute, Ottawa, Ontario K1H 8L1, Canada; Department of Biochemistry, Microbiology and Immunology, University of Ottawa, Ottawa, Ontario K1H 8M5, Canada; Centre for Infection, Immunity and Inflammation, University of Ottawa, Ottawa, Ontario K1H 8M5, Canada; Ottawa Institute of Systems Biology University of Ottawa, Ottawa, Ontario K1H 8M5, Canada; Apoptosis Research Centre, Children’s Hospital of Eastern Ontario Research Institute, Ottawa, Ontario K1H 8L1, Canada; Department of Biochemistry, Microbiology and Immunology, University of Ottawa, Ottawa, Ontario K1H 8M5, Canada; Centre for Infection, Immunity and Inflammation, University of Ottawa, Ottawa, Ontario K1H 8M5, Canada; Apoptosis Research Centre, Children’s Hospital of Eastern Ontario Research Institute, Ottawa, Ontario K1H 8L1, Canada; Centre for Infection, Immunity and Inflammation, University of Ottawa, Ottawa, Ontario K1H 8M5, Canada; Apoptosis Research Centre, Children’s Hospital of Eastern Ontario Research Institute, Ottawa, Ontario K1H 8L1, Canada; Department of Biochemistry, Microbiology and Immunology, University of Ottawa, Ottawa, Ontario K1H 8M5, Canada; Centre for Infection, Immunity and Inflammation, University of Ottawa, Ottawa, Ontario K1H 8M5, Canada; Ottawa Institute of Systems Biology University of Ottawa, Ottawa, Ontario K1H 8M5, Canada

**Keywords:** glioblastoma, immunotherapy, SMAC mimetic compounds, TGFβ, tumor-associated macrophages and microglia, anti-PD-1

## Abstract

**Background:**

Glioblastoma (GBM) is the most common primary brain tumor with an overall survival under 21 months. Despite extensive research effort, patient outcomes have improved minimally over the past several decades. The Inhibitor of Apoptosis (IAP) proteins are critical survival factors implicated in both immune regulation and gliomagenesis. Small molecule IAP antagonists called SMAC mimetic compounds (SMCs) are under investigation as cancer therapeutics across multiple malignancies, including GBM. SMCs induce GBM cell death in the presence of inflammatory cytokines, synergize with immune checkpoint inhibitors (ICI), and induce death of microglia and macrophages. Although SMCs show significant efficacy in murine models, complete eradication is not achieved. Here, we aimed to understand the limitations of SMCs in murine GBM and identify strategies to enhance efficacy of combination treatment with ICIs with the goal of informing future translational efforts.

**Methods:**

We use animal models, co-culture systems, flow cytometry, and multiplex immunohistochemistry to optimize SMC dosing and delivery, uncovering resistance mechanisms that address key unmet research needs.

**Results:**

We demonstrate that although GBM cells are immunologically recognizable, their location within the central nervous system (CNS) limits effective anti-GBM immunity following SMC and ICI combination therapy. Increasing SMC dose potently improves overall survival, which is associated with reduced intratumoral macrophage content, increased microglial involvement, and peripheral immunoactivation. Given the immunosuppressive role of TGFβ, the incorporation of TGFβ blockade further enhances survival outcomes.

**Conclusion:**

We comprehensively outline how SMCs can be used in conjunction with ICIs to treat GBM and propose strategies to maximize SMC efficacy.

Key Points Age and CNS location limit the efficacy of SMAC mimetic (SMC)-based immunotherapies for GBM.

Increasing the SMC dose reduces intratumoral macrophages and enhances the effectiveness of immune checkpoint therapy.

TGFβ is highly expressed in murine GBM and its blockade improves the efficacy of SMC-based immunotherapies.

Importance of the Study

GBM is the commonest and deadliest primary brain tumor. Patient prognoses have not improved in decades despite major advances in understanding GBM biology and the use of intensive, costly frontline treatments. Novel therapeutic strategies are desperately needed. SMAC mimetic compounds (SMCs) sensitize GBM cells to inflammatory cytokine-induced cell death, enhance anti-GBM immune responses, reduce intratumoral macrophage content, and extend survival in mouse models. Preclinical immunotherapy studies often rely on young immunocompetent mice with robust immune systems, GBM cell lines that are sensitive to SMCs *in vitro*, and GBM tumors with inflammatory microenvironments that differ substantially from human GBM—representing a ‘best case’ environment for therapeutic testing. Despite these favorable conditions, SMC and immune checkpoint inhibitor cotherapy does not consistently result in cures, highlighting the need for further optimization of treatments and novel combination strategies. We show that increasing the dose of SMCs and incorporating TGFβ blockade enhances therapeutic efficacy, leading to substantial cures in immunocompetent mouse models of GBM. These findings reveal key limitations of SMC-based immunotherapy for GBM and lay the groundwork for rational combination approaches and future translational studies.

Glioblastoma (GBM) is the commonest and deadliest primary brain tumor, most often seen in individuals between 50–70 years old, with increasing incidence despite few known risk factors^1–3^. Despite decades of intensive research into GBM biology, overall survival remains dismal—typically ranging from 16 to 21 months—and has not significantly improved^2^^,^^4–6^. GBM treatment is among the most expensive in oncology, involving maximal surgical resection followed by radiotherapy and temozolomide chemotherapy^7^. Therapeutic resistance in GBM is largely driven by significant intra- and intertumoral heterogeneity. Transformed versions of oligodendrocyte progenitor cells, radial glia, and neural progenitor cells coexist along gradients of inflammatory and metabolic reactivity^8^^,^^9^. Glioma stem cells, thought to be the cell of origin for GBM, are viewed as major factors in treatment resistance^10^ and exist along transcriptional gradients reminiscent of normal neuroinflammatory wound responses and neurodevelopment^11^.

GBM is an immunologically cold tumor^12^ characterized by an immunosuppressive microenvironment that induces T-cell exhaustion^13^^,^^14^ and promotes regulatory T-cell (Treg) phenotypes^15^. Transforming growth factor β (TGFβ) is a key cytokine within the GBM microenvironment^16^. TGFβ contributes to Treg recruitment and immunosuppressive activity^17–19^, including the recruitment and polarization of microglia and macrophages towards immunosuppressive states^20^^,^^21^. Tumor-associated microglia/macrophages (TAMs) are the dominant immune cell population in gliomas^22^. TAMs have key roles in all aspects of GBM biology, particularly in GBM progression and therapeutic resistance^22–26^, including suppression of T-cell infiltration and function^22^^,^^27^.

The Inhibitor of Apoptosis (IAP) proteins are a family of eight human anti-apoptotic proteins characterized by 1-3 baculovirus IAP-repeat (BIR) domains at their N-terminus^28^. The IAPs are essential for cell survival and immune function^29–31^, with aberrant expression associated with cancer. The BIR domains enable certain IAPs to directly inhibit caspases while several IAP members also possess an E3 ubiquitin ligase domain that can target proteins for degradation or for the formation of signaling complexes. Notably, cellular IAP 1 and 2 (cIAP1 and cIAP2; also known as *BIRC2* and *BIRC3*) and X-linked IAP (XIAP, also known as *BIRC4*) regulate survival signaling pathways such as Nuclear Factor-kappa B (NF-κB). The binding of Tumor Necrosis Factor alpha (TNF-α) to its receptor TNFR1 requires cIAP1/2 for activation of the classical NF-κB pathway, leading to expression of survival proteins^32–35^. The removal of cIAP1 and cIAP2 has 2 effects: converts TNF-α signalling from pro-survival to pro-death signals in cancer cells; and activates the alternative NF-κB pathway that leads to expression of genes involved in survival and immunity. In GBM, cIAP2 expression is significantly increased during gliomagenesis and under hypoxic conditions, contributing to malignancy and therapy resistance^36–38^. SMAC mimetic compounds (SMCs) have been developed to antagonize the IAPs, notably suppressing XIAP and inducing degradation of cIAP1 and cIAP2. SMCs have potent immunomodulatory effects by enhancing innate and adaptive immune responses^39^ and sensitizing cancer cells to inflammatory cytokine-induced cell death^40–49^, including GBM cells^50^.

SMCs have been demonstrated to synergize with immune checkpoint inhibitors in multiple cancer types and are currently under clinical investigation for various cancer indications. We previously demonstrated that combining SMCs with anti-programmed death 1 (α-PD-1) immune checkpoint blockade durably cures orthotopic murine GBM models in a T-cell dependent manner^51^. These preclinical murine models represent a ‘best case’ scenario for immunotherapy testing, using young immunocompetent mice with relatively inflamed GBM microenvironments and SMC sensitive GBM cells. Despite these favourable conditions, not all mice are cured with the cotherapy despite GBM cells remaining sensitive under typical microenvironment conditions, provided sufficient exposure to SMC and TNF-α^50^. Reasoning that suboptimal SMC delivery to the GBM tumor site contributes to therapeutic failure, we aimed to further our work in understanding resistance mechanisms in these immunocompetent ‘best-case’ murine models, explore the impact of SMC dose escalation on combination therapy efficacy, and characterize these dose effects on peripheral and tumor-associated immune cells.

## Methods

### Reagents

TNF-α (410-MT-010), IL-1β (401-ml) and TGFβ (7666-MB-005) were purchased from R&D systems. IFNγ was purchased from R&D systems (4875-M1) or Peprotech (315-05). IL-4 (214-14) was purchased from Peprotech. IL-10 (575806) was purchased from BioLegend. LPS (Tlrl-peklps) was purchased from Invivogen. GolgiPlug protein transport inhibitor (555029) was purchased from BD Biosciences. Ionomycin calcium salt was purchased from Millipore Sigma (P1585) or Life Technologies (I24222). PMA (P1585) was purchased from Millipore Sigma. LCL161 (S7009) was purchased from SelleckChem or provided by Novartis.

### Cell Culture

Cells were maintained at 37°C and 5% CO_2_ in DMEM media supplemented with 10% heat inactivated calf serum, 1% non-essential amino acids, and penicillin-streptomycin (Invitrogen). All cell lines were obtained from ATCC. T-cell: GL261 co-cultures were performed as previously described^52^. Briefly, 200 µL of peripheral blood was drawn from animals 7 days post-reimplantation. Red blood cells were lysed using ACK lysing buffer (A1049201 from ThermoFisher). GL261 cells were plated at 1 × 10^5^ cells/well in a 96 well plate and treated for 24 h with 75U IFNγ. Media was removed, cells were washed and isolated leukocytes were plated with monolayer GL261 cells in triplicate per animal. All wells were treated with GolgiPlug (1 µL/mL media). Negative control was naïve leukocytes. Positive control was treatment with PMA (20ng/mL) and Ionomycin (1 µg/mL). Following 4 h of co-culture or stimulation, cells were collected for flow cytometric analysis.

### Brain Tumor Models

All animal experiments were conducted with the approval of the University of Ottawa Animal Care and Veterinary Service (CHEO-3163). Female 6-week old C57BL/6 mice (obtained from Charles River Laboratories) were anesthetized with isofluorane and the surgical site prepared. 5 × 10^4^ CT2A or GL261 cells were implanted stereotactically over 1 minute in a 10 µL volume in the left striatum at coordinates: 0.5 mm anterior, 2 mm lateral from bregma, 3.5 mm deep. Skin was closed using surgical glue. An identical procedure was used for implantation of aged animals.

Mice were treated via oral gavage with either vehicle (30% 0.1 M HCl and 70% 0.1 M NaOAc pH 4.63) or LCL161 (75 mg/kg or 100 mg/kg) resuspended in 30% 0.1 N HCl and 70% NaOAc (pH 4.63).For treatment with immune checkpoint inhibitors, mice were treated intraperitoneally (i.p) with anti-PD-1 clone RMP1-14 (BE0146) or IgG2A isotype control (BE0089) from BioXcell. Treatments began Day 7 post-implantation. Gavage treatments were performed twice weekly over 2 weeks (Day 8, 10, 15, 17) unless otherwise stated. Checkpoint treatments occurred thrice weekly over 2 weeks (Day 7, 9, 11, 14, 16, 18). For experiments using anti-VEGFR2 (BioXcell, BE0060-25MG-A), treatments were 40 mg/kg initially followed by 20 mg/kg maintenance every 3–4 days over 2 weeks for a total of 3 treatments. For experiments using anti-TGFβ (BioXcell, BE0057), treatments were 5 mg/kg 3×/week for 2 weeks concurrent with anti-PD-1 treatments. Animal endpoint criteria include loss of >20% body weight, hunched posture, lethargy and significantly impaired ambulation.

### Microscopy

Mice were transcardially perfused on day 18 post-implant and brains formalin-fixed in 4% paraformaldehyde for 48 h. Fixed brains were processed for paraffin embedding and slicing (4 µm thick slices) at the Louise Pelletier Histology Core at the University of Ottawa. Slices were stained using the Akoya Opal polaris 7-color IHC detection kit (NEL811001KT) using previously established protocols^53^. Following deparaffinization and rehydration, slides underwent heat-induced antigen retrieval in pH6 or pH9 buffer (Akoya Biosciences, 1:10). Background autofluorescence was quenched using TrueBlack lipofuscin autofluorescence quencher (1:20, 10119-144, Biotium). Tissue was blocked using antibody diluent/block (Akoya, ARD1001EA) and incubated with primary antibodies. Slides were then incubated with Opal polymer HRP Ms+Rb (Akoya, ARH1001EA), and signals subsequently generated using Opal fluorophores in 1× Plus amplification diluent (FP1609). Primary antibodies used are listed in Table 1. Brains were stained with DAPI (0.5 µg/mL, PerkinElmer) and coverslipped using Dako fluorescence mounting medium (Agilent, S3023). Multiplex IHC images were acquired using the Zeiss AxioImager Z2 widefield microscope. Images were spectrally unmixed in Zeiss Zen Black software using single stained controls. Unmixed images were analyzed using QuPath software.

**Table 1:** Antibodies used in flow cytometry and immunohistochemistry

Target	Conjugate (flow) or antigen retrieval pH (IHC)	Manufacturer	Catalogue #
TruStain FcX (anti-mouse CD16/32)		BioLegend	101320
Zombie Violet Fixable Viability Kit	BV421 Equivalent	BioLegend	423114
Zombie Green Fixable Viability Kit	AF488/GFP Equivalent	BioLegend	423112
Anti-mouse phosphatidylserine	AF488	Sigma Aldrich	16-256
Anti-mouse calreticulin	PE	Novus	NBP1-47518PE
Anti-mouse IFNγ	PE	BioLegend	505808
Anti-mouse TNF-α	APC	BioLegend	506308
Anti-mouse IDO1	AF647	BioLegend	654004
Anti-mouse KLRG1	FITC	BioLegend	138410
Anti-Mouse MHCI (H-2Kd/H-2Dd)	AF647	BioLegend	114712
Anti-Mouse MHCII (I-A/I-E)	BV605	BioLegend	107639
Anti-mouse Qa-1b	BV786	BD Biosciences	744390
Anti-mouse Qa-2	FITC	BioLegend	121710
Anti-Mouse CCR7	BV605	BioLegend	120125
Anti-mouse CTLA4	BV605	BioLegend	106323
Anti-mouse PD-L2	APC	BioLegend	107210
Anti-mouse PD-L1	BV421	BioLegend	124315
Anti-mouse CD86	BV786	BioLegend	105043
Anti-mouse CD80	AF488	BioLegend	104716
Anti-mouse Galectin-9	APC	BioLegend	136110
Anti-mouse OX40	PE	BioLegend	119409
Anti-mouse Ki67	BV711	BD Biosciences	563755
Anti-mouse PD-1	BV605	BioLegend	135220
Anti-mouse LAG3	BV711	BioLegend	125243
Anti-mouse TIM3	PE	BioLegend	119703
Anti-mouse Ly6C	AF488	BioLegend	128022
Anti-mouse Ly6G	BV786	BioLegend	127645
Anti-mouse F4/80	AF700	BioLegend	123130
Anti-mouse F4/80	APC	eBioscience Thermofisher	17-4801-80
Anti-mouse CD3	BV786	BioLegend	100232
Anti-mouse CD3	APC/Cy7	BioLegend	100222
Anti-mouse CD3	Pacific Blue	BioLegend	100214
Anti-mouse CD3	FITC	BioLegend	100306 & 100204
Anti-mouse CD4	AF700	BioLegend	100536
Anti-mouse CD8	FITC	BioLegend	100706
Anti-mouse CD8	FITC	Invitrogen	MA5-16759
Anti-mouse CD11b	APC	BioLegend	101212
Anti-mouse CD11b	PE	BioLegend	101207
Anti-mouse CD25	APC	eBioscience	17-0251-81A
Anti-mouse CD45	BV786	BioLegend	103149
Anti-mouse CD45	PE	BioLegend	147712
Anti-mouse CD69	BV605	BioLegend	104529
Anti-mouse CD86	PE	BioLegend	105007
Anti-mouse CD127	AF488	BioLegend	135018
Anti-mouse TMEM119	1:250, AR pH6	ThermoFisher	PA5-119617
Anti-mouse IBA-1	1:200, AR pH9	ThermoFisher	MA5-16363
Anti-mouse Arginase-1	1:100, AR pH9	ThermoFisher	PA5-85267
Anti-mouse CD3	1:100, AR pH9	Abcam	ab16669
Anti-mouse CD8	1:1500, AR pH9	Abcam	ab217344
Anti-mouse TIM3	1:500, AR pH9	Cell Signaling	83822S
Anti-mouse PD-1	1:200, AR pH9	Cell Signaling	84651S
Anti-mouse OX40	1:50, AR pH6	Cell Signaling	61637S
Anti-mouse Cleaved Caspase-3	1:100, AR pH9	Cell Signaling	9661S
Anti-mouse CD86	1:500, AR pH9	Cell Signaling	19589S
Anti-mouse CD206	1:500, AR pH9	Cell Signaling	24595S

### Western Blotting

Cells were lysed in RIPA lysis buffer containing protease inhibitor cocktail (Roche). Equal amounts of soluble protein were separated on polyacrylamide gels followed by transfer to nitrocellulose membranes. Total protein was quantified using Revert 700 total protein stain (LI-COR Bio, 926-11011) and ImageStudio software (LI-COR Bio). Individual proteins were detected using antibodies for cIAP1/2 (1:1000, CY-P1041, Cyclex) and XIAP (1:1000, M044-3, MBL Life Science). Full blots (Supplemental Fig. 6) were cropped as indicated (red boxes) for display in main figures (Fig. 6F).

### Cytokine Expression Profile

Cell supernatants were collected 24 h post-treatment with LCL161 (10 µM) or matched vehicle. Media was concentrated using Amicon Ultra-4 Centrifugal Filters (UFC801096, Millipore) and TGFβ1-3 levels were analyzed using the TGFβ assays at Eve Technologies.

### Flow Cytometry

All antibodies and associated details are listed in Table 1. Analyzed organs were collected on day 11 (*in vivo* TGFβ treated) or day 18 (all other *in vivo* flow) post-implant. Tissues were dissociated using gentleMACS dissociator (130-096-427) from Miltenyi Biotec. Resulting suspensions were strained through 70 µm cell strainers (352350, Corning) prior to counting and normalization of populations across analyzed groups. Cells were stained with Fc block (anti-CD16/CD32) and Zombie viability dyes at 1:300 dilution prior to surface stains. Cells were fixed using intracellular fixation solution (ThermoFisher/eBioscience, 88-8824-00). If intracellular targets were being analyzed, cells were permeabilized using permeabilization buffer as per manufacturers protocol. Intracellular targets were stained same day as analysis. Antibody stains were performed at 1:200 dilution unless otherwise indicated. Cells were analysed on a BD Fortessa (BD Biosciences) flow cytometer. Data was analysed using FlowJo software (BD Biosciences). Compensation was performed using UltraComp eBeads (01-222-42) from ThermoFisher/Invitrogen.

### Statistical Analysis

Comparison of Kaplan-Meier survival plots was conducted by log-rank analysis and subsequent pairwise multiple comparisons were performed using the Holm-Sidak method (GraphPad). Comparison between multiple treatment groups was analysed using one- or two-way ANOVA followed by post hoc analysis using Tukey’s multiple comparison test (GraphPad). Comparison of treatment pairs was analysed by two-sided t-tests (GraphPad).

## Results

### Murine GBM Cells Express Factors Involved in Antigen Presentation and T-Cell Recognition

**Figure 1:**
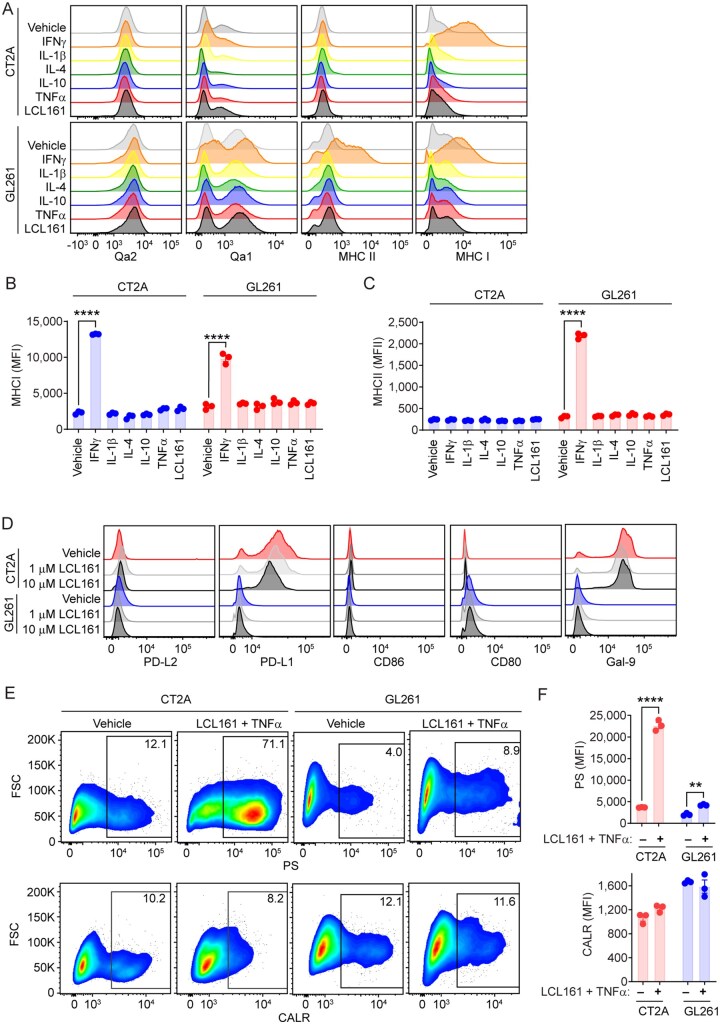
**Murine GBM cells express immune proteins supporting immune recognition**. A) Flow cytometric analysis of Qa2 (FITC), Qa1 (BV786), MHCII (BV605), and MHCI (AF647) expression on CT2A and GL261 cells treated with IFNγ (50U), IL-1β (20ng/mL), IL-4 (20ng/mL), IL-10 (20ng/mL), TNF-α (10ng/mL), vehicle (DMSO), or LCL161 (10µM). B, C) Mean fluorescent intensity (MFI) of (B) MHCI and (C) MHCII in response to treatments in CT2A and GL261 cells. N = 3 for all treatment groups. ****P < 0.0001 by two-way ANOVA using Tukey’s HSD multiple comparison test. D) Flow cytometric analysis of PD-L2 (APC), PD-L1 (BV421), CD86 (BV786), CD80 (AF488), and Galectin-9 (APC) expression on CT2A and GL261 cells treated with vehicle or LCL161 (1µM or 10µM) for 24 h. E, F) Flow cytometric analysis of phosphatidylserine (PS; AF488) and calreticulin (CALR; PE) expression on CT2A and GL261 cells treated with 10µM LCL161 and 10ng/mL TNF-α. Bar plots show MFI of PS and CALR expression. N = 3 per treatment group. **P < 0.01; ****P < 0.0001 by two-way ANOVA using Tukey’s HSD multiple comparison test.

We first investigated whether SMCs or cytokines affect the expression of immunomodulatory proteins that facilitate T-cell activation and recognition, reasoning that increased SMC dosing and their consequent pro-inflammatory effects may alter the expression of molecules relevant to anti-GBM immunity. To determine the basal and inducible expression levels of immunomodulatory molecules, murine CT2A and GL261 GBM cells were stained for MHCI, MHCII, Qa1 (mouse HLA-E homolog) and Qa2 (mouse HLA-G homolog) following treatment with the SMC LCL161 and various cytokines. Qa1 and Qa2 expression remained unchanged across cytokine treatment (Fig. 1A). MHCI expression was increased in both CT2A and GL261 cells following IFNγ treatment, while MHCII expression was induced only in GL261 cells (Fig. 1A–C). LCL161 did not affect the expression of immune checkpoint ligands (Fig. 1D); in this context, CT2A cells exhibited higher baseline levels of PD-L1 and Galectin 9 (Gal-9) compared to GL261. We next examined the expression levels of surface markers associated with phagocytosis of dying cells. Cell death was induced by combined LCL161 and TNF-α treatment and the cells were stained for the damage associated molecular patterns phosphatidylserine (PS) and calreticulin (CALR). Both CT2A and GL261 cells showed significant increases in expression of PS following treatment with LCL161 and TNF-α (Fig. 1E, F top) while CALR expression remained unchanged (Fig. 1E, F bottom). These findings indicate that murine GBM cells express markers that support phagocytosis, T-cell recognition, and immune checkpoint mediated inhibition of T-cell cytotoxicity. Thus, resistance to SMC-based immunotherapies is unlikely to stem from a lack of immunomodulatory protein expression or compensatory treatment-induced changes that enable immune evasion.

### The CNS Environment Uniquely Limits Primary but Not Memory Anti-GBM Immune Responses

**Figure 2:**
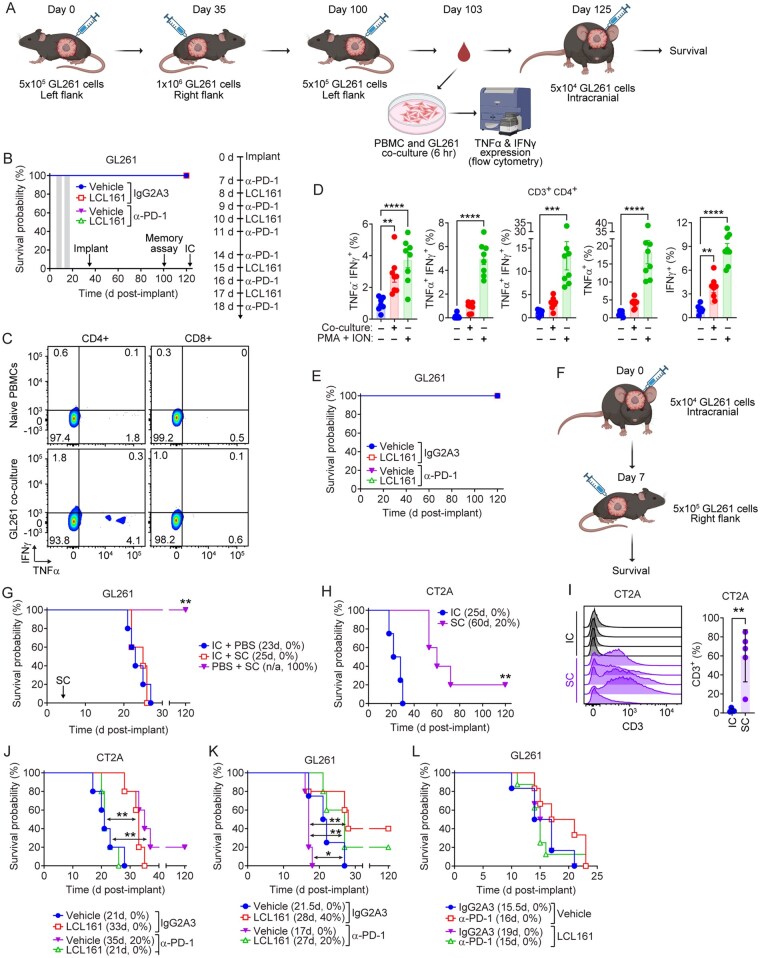
**CNS location restricts primary but not memory anti-GBM immune responses**. A) Experimental outline for subcutaneous (SC) GL261 tumor model and subsequent analysis. Mice were implanted with 5 × 10^5^ GL261 cells SC and treated orally with vehicle or 75mg/kg LCL161 and intraperitoneally with 10mg/kg αPD-1 or isotype control per indicated schedule (B, right). On post-implantation day 35 mice were implanted with 1 × 10^6^ GL261 cells in opposite flank. On post-implantation day 100, mice were implanted with 5 × 10^5^ GL261 cells SC. Peripheral blood was drawn 3 days later. On post-implantation day 125, mice were implanted with 5 × 10^4^ GL261 cells intracranially (IC). N = 5 per treatment group. B) Data represent Kaplan-Meier curve depicting mouse survival following SC implant. C, D) Flow cytometric analysis of IFNγ (PE) and TNF-α (APC) expression by CD3^+^ CD4^+^ cells following 6-hour co-culture with GL261 cells or treatment with PMA and ionomycin (ION). Bar plots show the percentage of CD3^+^ CD4^+^ cells expressing TNF-α and IFNγ. N = 8 for all treatment groups. **P < 0.01; ***P < 0.001; ****P < 0.0001 by one-way ANOVA using Tukey’s HSD multiple comparison test for (D). E) Kaplan-Meier curve depicting mouse survival following IC implant indicated in (A). No further treatments were administered. F) Experimental outline for sequential SC and IC implantation of GL261 cells. Mice were implanted with 5 × 10^4^ GL261 cells IC. At 7 days post-implantation, mice were implanted with 5 × 10^5^ GL261 cells SC. N = 5 per treatment group. G) Data represent Kaplan-Meier curve depicting mouse survival. Statistical analysis by log-rank with Holm-Sidak multiple comparison. N = 5 per treatment group. **P < 0.01. H) Mice were implanted with 5 × 10^4^ CT2A cells IC or 5 × 10^5^ SC. Data represent Kaplan-Meier curve depicting mouse survival. Log-rank with Holm-Sidak multiple comparison. N = 5 per treatment group. **P < 0.01. I) Flow cytometric analysis of CD3 (APC-Cy7) expression in IC and SC CT2A tumors at endpoint. Bar plots represent percent of total live cells that are CD3^+^. N = 5 per indicated implant site. **P < 0.01 by unpaired t-test. J) Middle aged (10–11-month-old) mice were implanted with 5 × 10^4^ CT2A or (K) GL261 cells and treated orally with 75mg/kg LCL161 and intraperitoneally with 10mg/kg αPD-1 or isotype control. Data represent Kaplan-Meier curves depicting mouse survival. Log-rank with Holm-Sidak multiple comparison. N = 5 per treatment group. *P < 0.05; **P < 0.01. L) Old (18-month-old) mice were implanted with 5 × 10^4^ GL261 cells and treated orally with LCL161 or vehicle and αPD-1 or isotype control. Data represent Kaplan-Meier curves depicting mouse survival. Log-rank with Holm-Sidak multiple comparison. N = 5 per treatment group. Values in legends of Kaplan-Meier curves represent median survival (days) and overall survival (percentage).

We next evaluated the role of the CNS microenvironment in modulating the efficacy and resistance to SMC and immune checkpoint combination therapy for GBM. We focused on the GL261 model given the greater resemblance of the GL261 immune microenvironment to the human condition compared to the CT2A model^54^. We first assessed immune responsiveness outside of the immune-restricted CNS, wherein we implanted GL261 cells subcutaneously (SC) in the left flank and treated the mice with LCL161 and α-PD-1 (Experiment outline Fig. 2A). No visible tumors developed within 35 days post-implantation regardless of treatment (Fig. 2B). Subsequent reinjection of GL261 in the opposite flank resulted in no tumor growth, suggesting immune-mediated rejection. At day 100 post-initial implantation, mice were rechallenged with GL261 cells and peripheral blood mononuclear cells (PBMCs) were collected for *ex vivo* coculture with GL261 cells. We evaluated the expression of key effector cytokines TNF-α and IFNγ from activated T-cells, reasoning that memory T-cell recognition of GL261 antigens would enhance expression of either cytokine. Treatment with phorbol 12-myristate 13-acetate (PMA) and ionomycin was used as a positive control. Only CD4^+^ T-cells showed increased IFNγ expression (Fig. 2C, D), consistent with inducible MHCII expression by GL261 cells (Fig. 1C). No significant changes were observed in CD8^+^ T-cell expression of TNF-α or IFNγ (Supplemental Fig. 1).

To assess whether this peripheral immune memory would result in rejection of brain GBM tumors, we implanted GL261 cells intracranially (IC) into the orthotopic environment. All implanted animals cleared the tumor and survived long term without additional treatment (Fig. 2E). To determine whether clearance of a subcutaneous tumor leads to an abscopal effect-type clearance of a concomitant intracranial tumor, GL261 cells were implanted intracranially followed by subcutaneous implantation 7 days later (Fig. 2F). All subcutaneous tumors were cleared regardless of the presence of an intracranial tumor. However, all animals with an intracranial tumor reached endpoint within 30 days post-implantation (Fig. 2G), suggesting the CNS location uniquely restricts primary but not memory anti-GL261 immune responses within the ∼30-day survival window. Unlike GL261, CT2A tumors grew robustly when implanted subcutaneously (Fig. 2H) and exhibited higher CD3^+^ T-cell infiltration relative to their intracranial CT2A tumor counterparts (Fig. 2I). Therefore, murine GBM cells express immunogenic antigens allowing for immune recognition, clearance and robust memory responses. The CNS environment uniquely limits primary T-cell anti-GBM immunity and consequently the efficacy of SMC and immune checkpoint inhibitor cotherapy.

### Age-Related Changes Limit the Efficacy of SMC-Based Immunotherapy

As GBM mostly affects individuals aged 50–70^55^, and most preclinical studies use ∼8 week old mice (equivalent to young adult humans), we evaluated the efficacy of the combination immunotherapy in aged animals to better reflect the clinical context. Mice aged 10 months (corresponding to middle age humans^56^) were implanted intracranially with GL261 or CT2A cells and treated as before. In CT2A-bearing mice, the efficacy of cotherapy was abolished (Fig. 2J) whereas long-term survivors were still observed in GL261-bearing mice (Fig. 2K). The best response was observed in GL261-bearing mice treated with LCL161 alone, and median survival of CT2A-bearing mice was also increased relative to vehicle controls. To investigate age-associated immunosuppressive mechanisms, endpoint animals were analyzed for expression of IDO and KLRG1. Both factors were significantly increased in immune cells within the tumor microenvironment with no treatment-dependent differences (Supplemental Fig. 2A, B). When GL261 cells were implanted in 18 month old mice (corresponding to ‘old’ humans^56^), the efficacy of all treatments was abolished (Fig. 2L), suggesting age-related changes in the CNS and immune system significantly impair therapeutic efficacy. These findings illustrate the need to optimize SMC dosing and explore additional immunotherapeutic combinations for successful clinical translation.

### SMCs Increase Intratumoral Microglial Content While Reducing Infiltration of Peripheral Macrophages

**Figure 3:**
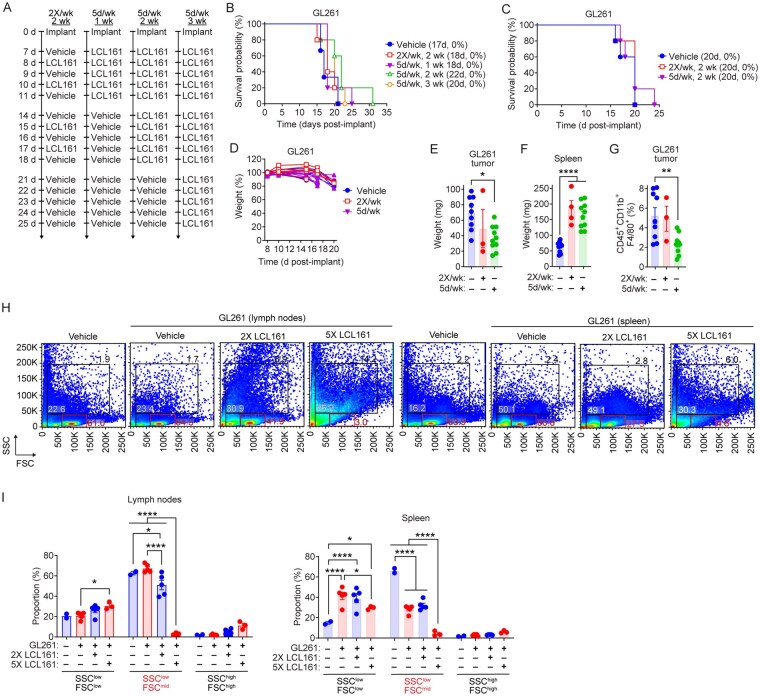
**High dose SMC is well tolerated and reduces tumor burden and TAMs**. A) Treatment schedules for LCL161 dose-escalation. B) Mice were implanted with 5 × 10^4^ GL261 cells and treated orally with vehicle or 75mg/kg LCL161 as in (A). N = 3 for vehicle, N = 5 for all other treatment groups. C) Mice implanted with GL261 cells were treated with vehicle or 100mg/kg LCL161 2×/week or 5×/week for 2 weeks. N = 5 per treatment group. Data represent the Kaplan-Meier curve depicting mouse survival. Log-rank with Holm-Sidak multiple comparison. Values in legends of Kaplan-Meier curves represent median survival (days) and overall survival (percentage). D) Mouse weights during the course of 100 mg/kg LCL161 treatment (as in C). E, F) Weights of mouse spleens and resected tumor bulk following 100mg/kg LCL161 treatments described in (C). G) Flow cytometric analysis of CD45 (BV786) and CD11b (APC) expression among live cells in GL261 tumors at end of LCL161 treatment schedule. Bar plots show the percent of live cells positive for CD45, CD11b, and F4/80 (PE). N = 9 for vehicle, N = 3 for 2×/week and N = 10 for 5×/week treatment groups. *P < 0.05; **P < 0.01; ****P < 0.0001 via one-way ANOVA using Tukey’s HSD multiple comparison test. H, I) Flow cytometric analysis of side scatter (SSC) and forward scatter (FSC) of lymph nodes and spleen from GL261-bearing mice following two-week treatments as in (A, C, D). Bar plots are proportion of cells within indicated gates and tissues. N = 2 for naïve, N = 3 for 5×/week, N = 5 per remaining treatment group. *P < 0.05; ****P < 0.0001 via two-way ANOVA using Tukey’s HSD multiple comparison test.

Given that a high dose of SMC can induce GBM cell death via interactions among microglia, macrophages and astrocytes^50^, we assessed whether increasing the dose and frequency of LCL161 administration would improve survival in mice bearing intracranial GL261 tumors. LCL161 was administered orally at 75 mg/kg daily for one, two or three weeks, compared to the twice weekly regimen over a two-week period (Fig. 3A). No survival benefit was observed with increased frequency (Fig. 3B). To assess the impact of dose escalation, LCL161 was administered at 100 mg/kg either twice weekly (2×/wk) or every day for 2 weeks (5×/wk). No survival benefit was noted (Fig. 3C) and the increased dose was well tolerated with no adverse changes to mouse weight (Fig. 3D). Despite the lack of survival improvement, the high-dose/high-frequency treatment reduced the overall size of resectable tumors (Fig. 3E) and increased spleen size (Fig. 3F), consistent with increased cell numbers and enlarged spleens observed in cIAP1 and XIAP deficient mice^57^. Intratumoral macrophages and microglia were reduced only in animals treated in the high-dose/high-frequency group as measured by the number of CD45, CD11b, and F4/80 positive cell counts (Fig. 3G). Lymph nodes and spleens from these mice showed significant reductions in the SSC^low^ FSC^mid^ populations (Fig. 3H, I), indicating lymphocyte depletion with high-dose/high-frequency treatment with SMCs.

**Figure 4:**
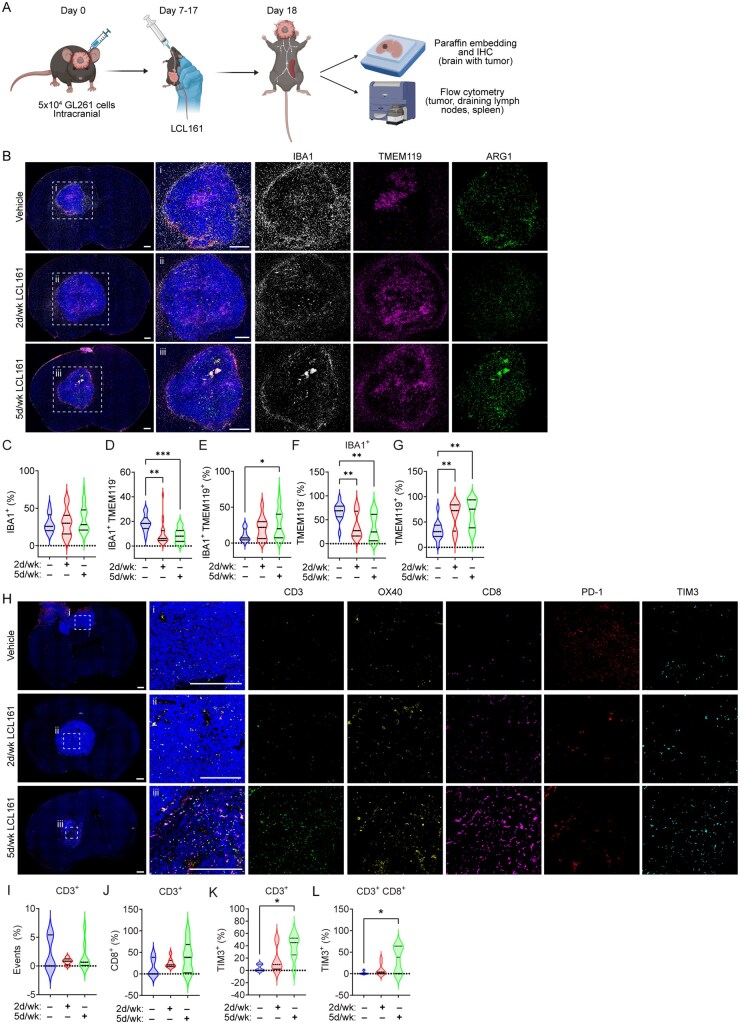
**High dose SMC remodels the GBM microenvironment**. A) Experimental outline for IHC and flow cytometric analysis of IC GL261 tumors following high dose LCL161 treatments. Mice bearing IC GL261 tumors were treated orally 2×/week or 5×/week for two weeks with 100mg/kg LCL161. B) At day 18 post-implant, brains were processed for immunohistochemical staining of IBA1, TMEM119, and Arginase-1 (ARG1). Scale bar: 500 µm. C–G) Total IBA1^+^ cells (C) and TMEM119 subsets (D–G) within identified tumor margins were quantified and plotted as a percentage of total DAPI detections or as a fraction of total IBA1^+^ cells. H) Mice bearing IC GL261 tumors were stained for CD3, CD8, PD-1, TIM3 and OX40 expression. I–L) Total CD3^+^ cell counts, CD8^+^ subsets, and expression of TIM3 were quantified and plotted as either percent of total DAPI detections within tumor margins or as a proportion of total CD3+ events. N = 2 vehicle, N = 4 2d/wk, N = 5 5d/wk LCL161 treated animals per group *P < 0.05; ***P < 0.001; via one-way ANOVA using Tukey’s HSD multiple comparison test.

To evaluate tumor-specific effects of increased dose and frequency of SMC treatment, we analyzed tumor-bearing mouse brains for TAMs and infiltrating T-cells (Fig. 4A). We previously demonstrated that SMCs induce microglia and macrophage death, consequently secreting factors that lead to the eradication of GBM cells in the presence of a SMC^50^. Total intratumoral IBA1 content, representing all TAMs, was not significantly affected by LCL161 treatment (Fig. 4B, C). However, daily treatments (5×/wk) with LCL161 significantly increased the proportion of TMEM119 positive microglia (Fig. 4D, F) and both treatment schedules reduced TMEM119 negative macrophages (Fig. 4E, G)^58^, suggesting a shift in TAM composition. No changes were observed in overall cleaved caspase-3 (CC3) levels (Supplemental Fig. 3A). Both proinflammatory (CD86+) and anti-inflammatory (CD206+) macrophages, as well as inflammatory microglia, were significantly reduced (Supplemental Fig. 3B), consistent with prior findings that macrophages and polarized inflammatory microglia are more sensitive to SMC-induced cell death^50^^,^^59^. The analysis of T-cell populations revealed no significant changes in total CD3+ cells or T-cell subsets (Fig. 4I, J; Supplemental Fig. 3C). Expression of the T-cell activation marker OX40 and immune checkpoint PD-1 were also unchanged following SMC treatment (Supplemental Fig. 3D, E). However, the daily 5×/wk treatment significantly increased TIM3 checkpoint expression among CD3^+^ cells, especially among the CD8^+^ fraction (Fig. 4K, L).

### SMC Treatment Enhances Peripheral T-Cell Activation but Not Intratumoral T-Cell Responses

**Figure 5:**
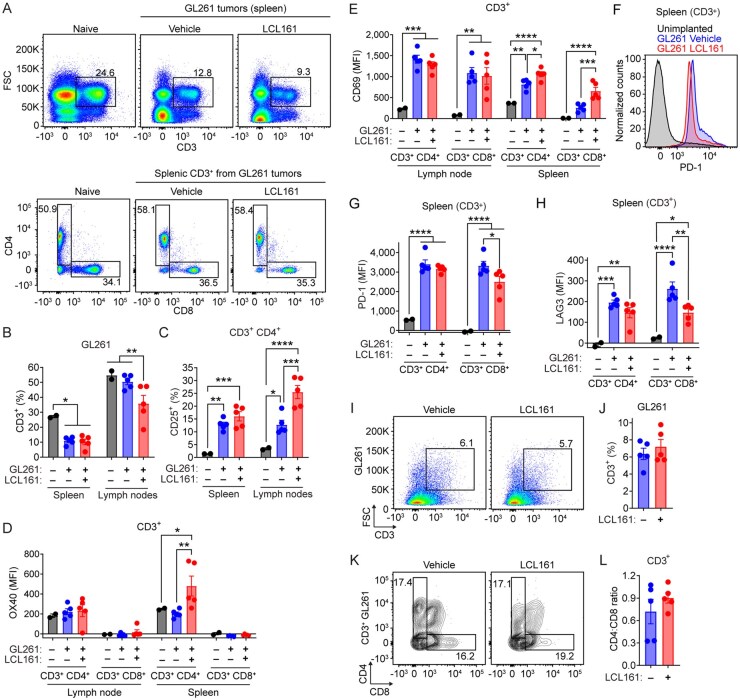
**SMCs upregulate T-cell activation markers in peripheral lymphoid organs but not in brain tumors**. A) Flow cytometric analysis of CD3 (BV786), CD4 (AF700) and CD8 (FITC) in spleen from mice bearing GL261 tumors and treated with vehicle or 100mg/kg LCL161 2d/week. B) Quantification of live cells CD3^+^ in spleen and lymph nodes across indicated treatment groups. N = 2 for unimplanted, N = 5 per remaining treatment groups. *P < 0.05; **P < 0.01 via two-way ANOVA using Tukey’s HSD multiple comparison test. C) Proportion of CD3^+^CD4^+^ cells in indicated organ expressing CD25 receiving indicated treatments. D, E) MFI of OX40 (D) and CD69 (E) on CD3^+^CD4^+^ and CD3^+^CD8^+^ cells in indicated organ receiving indicated treatment. F) PD-1 expression of splenic CD8+ T-cells from mice with or without IC GL261 tumors, quantified in (G). H) MFI of LAG3 expression in CD3^+^ cells in spleen. *P < 0.05; **P < 0.01; ***P < 0.001; ****P < 0.0001 via two-way ANOVA using Tukey’s HSD multiple comparison test. I–L) Flow cytometric analysis of CD3 positivity and subsets in resected GL261 tumors following treatment with vehicle or 100mg/kg LCL161 2d/wk. J) Quantification of proportion of live cells CD3^+^. L) Ratio of CD4: CD8 cells among CD3^+^ populations.

As SMCs are delivered orally and their effects are therefore systemic, we aimed to further characterize peripheral immune responses. We assessed the effects of increased SMC dosing on immune cells from the spleen and draining lymph nodes. High frequency (5×/wk) LCL61 treatments were excluded due to lymphocyte depletion (Fig. 3H, I). Intracranial GL261 tumors significantly reduced splenic T-cell numbers (Fig. 5A, B), specifically among the CD8^+^ population (Supplemental Fig. 4A). LCL161 treatment also reduced T-cell counts in the draining cervical lymph nodes (Fig. 5B). Intracranial GL261 tumors increased the expression of the chemokine receptor CCR7, memory and effector T-cell marker CD127, and immune checkpoint CTLA-4 on CD4^+^ T-cells in both spleen and lymph nodes, with LCL161 having no impact on expression levels (Supplemental Fig. 4B, C). In contrast, LCL161 significantly increased expression of CD25 (IL-2R), OX40, and CD69 on CD4^+^ T-cells (Fig. 5C-E) in both lymph nodes and spleen. Splenic PD-1 expression was significantly increased following tumor implantation (Fig. 5F, G), and LCL161 slightly reduced PD-1 and LAG3 expression on CD8^+^ T-cells (Fig. 5H). Altogether, these results suggest that SMC treatment in mice bearing intracranial GL261 tumors increases expression of T-cell activation markers and decreases expression of immune checkpoints in peripheral lymphoid organs.

We next assessed whether peripheral immunoactivation extended to the tumor microenvironment. Consistent with our immunohistochemistry findings (Fig. 4), no changes were observed in total T-cell infiltrates, CD4^+^ T-cell proportions (Fig. 5I-L), expression of activation markers (Supplemental Fig. 4D, E), or immune checkpoint expression (Supplemental Fig. 4F–H) following 2×/wk treatments with 100 mg/kg LCL161. Together, these data suggest increased SMC dosing enhances peripheral immune activation and shifts TAM composition towards microglia within GL261 tumors but has minimal impact on intratumoral T-cell populations. These findings demonstrate that intracranial tumors upregulate the expression of immune checkpoints in peripheral lymphoid organs. Despite substantial T-cell activation following SMC treatment, the immunosuppressive mechanisms in CNS lesions are resistant to this immunostimulation alone. This supports the rationale for combining SMCs with PD-1 blockade to remove immunosuppressive hurdles and achieve synergistic anti-GBM effects as previously demonstrated^50^^,^^51^. This also suggests that targeting multiple immunosuppressive pathways is necessary for effective anti-tumor responses in the CNS.

### SMC Dose and TGFβ Are Key Limitations for α-PD-1 Based Cotherapy

**Figure 6:**
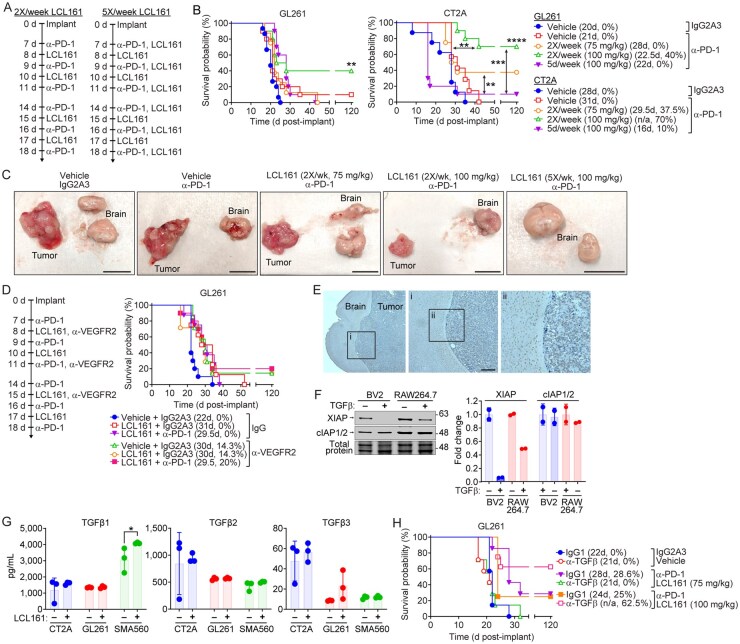
**Increased SMC dose and TGFβ blockade enhance α-PD-1 efficacy in GBM**. A, B) Mice were implanted with 5 × 10^4^ GL261 or CT2A cells and treated orally with 75mg/kg or 100mg/kg LCL161 and intraperitoneally with 10mg/kg of isotype control or α-PD-1 antibody as per schedule in (A). B) Data represent the Kaplan-Meier curve depicting mouse survival. Log-rank with Holm-Sidak multiple comparison. Left, GL261: N = 15 Vehicle/IgG2A3, N = 8 Vehicle/α-PD-1, N = 6 2×/week (100mg/kg)/α-PD-1, N = 10 5×/week (100mg/kg)/α-PD-1, N = 8 2×/week (75mg/kg)/α-PD-1. Right, CT2A: N = 8 Vehicle/IgG2A3, N = 8 2×/week (75mg/kg)/α-PD-1, N = 10 remaining treatment groups. *P < 0.05; **P < 0.01; ***P < 0.001; ****P < 0.0001. C) Representative images of endpoint mouse brains and CT2A tumors in animals receiving indicated treatments. Scale bar: 1cm. D) Mice were implanted with 5 × 10^4^ GL261 cells and treated orally with 100mg/kg LCL161 or vehicle, intraperitoneally with 10mg/kg α-PD-1 or isotype control, and α-VEGFR2 or isotype control as per indicated schedule. Data represent Kaplan-Meier curve depicting mouse survival. Log-rank with Holm-Sidak multiple comparison. N = 10 for Vehicle + IgG2A3 + IgG1 and LCL161 + α-PD-1 + α-VEGFR2. N = 8 for LCL161 + IgG2A3 + IgG1 and LCL161 + α-PD-1 + IgG1. N = 7 for Vehicle + IgG2A3 + α-VEGFR2. N = 6 for LCL161 + IgG2A3 + α-VEGFR2. E) Representative H&E and TGFβ immunohistochemical staining of mouse brain bearing intracranial GL261 tumor. Scale bar: 250µm. Brown represents positive TGFβ signal. F) Western blot analysis of BV2 mouse microglia and RAW 264.7 mouse macrophages treated with vehicle or 20ng/mL TGFβ for 24h and assayed for cIAP1/2 and XIAP expression levels. G) Expression of TGFβ1-3 isoforms in culture media from murine GBM cell lines treated with vehicle or LCL161 (10µM). H) Mice were implanted with 5 × 10^4^ GL261 cells and treated with 75 or 100mg/kg LCL161 and 10mg/kg α-PD-1 and α-TGFβ antibodies or vehicle and isotype control as indicated. Data represent the Kaplan-Meier curve depicting mouse survival. Log-rank with Holm-Sidak multiple comparison. N = 7 or 8 per treatment group where indicated. *P < 0.05; **P < 0.01; ***P < 0.001. Values in legends of Kaplan-Meier curves represent median survival (days) and overall survival (percentage). **P < 0.01; ****P < 0.0001 via two-way ANOVA using Tukey’s HSD multiple comparison test.

To assess how increased SMC dosing and frequency affect the efficacy of combination therapy with α-PD-1 checkpoint blockade, we treated mice bearing intracranial GL261 or CT2A tumors with escalating doses of the SMC LCL161 (Fig. 6A). Increasing the 2×/wk LCL161 dose from 75 mg/kg to 100 mg/kg significantly enhanced therapeutic efficacy in both tumor models (Fig. 6B), with 70% of CT2A-bearing animals surviving long term. In contrast, daily (5×/wk) LCL161 treatment significantly reduced overall survival in both tumor models, most notably in CT2A, where the number of long-term survivors was halved relative to the 2×/wk 75 mg/kg regimen. Among endpoint animals treated with the 5×/wk regimen, only 2 out of 9 brains had observable tumors (Fig. 6C, Table 2), suggesting off-tumor toxicity consistent with reduced lymphocyte populations (Fig. 3H, I). Cytokine release syndrome, a known dose-limiting toxicity in LCL161 clinical trials^60–62^, was not assessed but may explain the observed reductions in survival. It is clear across both models that increased SMC dosing significantly improves cotherapy efficacy, achieving near complete cures in the CT2A model.

**Table 2:** Endpoint outcomes following treatment of mice bearing intracranial CT2A tumors

Treatment group	Survivors/total	Proportion with resectable tumors at endpoint
Vehicle + IgG2A3	0/9	9/9 (100%)
Vehicle + αPD-1	0/10	10/10 (100%)
LCL161 (2d/wk, 75mg/kg) + αPD-1	3/8	5/5 (100%)
LCL161 (2d/wk, 100mg/kg) + αPD-1	7/10	3/3 (100%)
LCL161 (5d/wk, 100mg/kg) + αPD-1	1/10	2/9 (22%)

Despite the significant improvements in survival, the GL261 model remained relatively refractory. Given that 100 mg/kg is the maximum feasible dose due to LCL161 solubility, animal care volume limits, and that increased SMC frequency reduced survival, we sought additional combinations to further enhance efficacy in the intracranial GL261 model.

We previously showed that hypoxia and TGFβ protect microglia and macrophages from SMC-mediated cytotoxicity *in vitro*^50^. Accordingly, we tested whether targeting either of these GBM hallmarks would improve outcomes. We first aimed to normalize tumor vasculature using low dose VEGFR2 blockade, which restores normal blood flow and thereby reoxygenates the tumor^63^^,^^64^ (Fig. 6D, left). The beneficial effects of VEGFR2 blockade can be related to reducing edema^65^, a contributor to GBM symptoms and pathology^66^. α-VEGFR2 treatment in mice bearing orthotopic GL261 tumors did not improve mouse survival when combined with α-PD-1 and high dose LCL161 (Fig. 6D, right), suggesting hypoxia and tumor vasculature are not limiting factors for SMC and α-PD-1 cotherapy.

TGFβ is secreted by both GBM cells and TAMs, and plays a major role in GBM progression^67–70^, monocyte and microglia homeostasis^71^^,^^72^, and immunosuppression within the GBM tumor microenvironment^73^^,^^74^. Immunohistochemistry revealed strong expression of TGFβ at the tumor margins and within tumors in orthotopic GL261 tumors (Fig. 6E), consistent with prior findings^17^^,^^75^^,^^76^. We previously showed that TGFβ reduces microglia and macrophage sensitivity to SMC-mediated cell death, an effect not replicated by treatment with IL-4 or IL-10^50^. Given that much of this protection is ablated following treatment with the more potent dimeric SMC compound AZD5582^50^, we assessed whether TGFβ treatment affected IAP levels in BV2 immortalized microglia and RAW264.7 immortalized macrophages. Interestingly, despite TGFβ conferring reduced sensitivity of BV2 and RAW246.7 cells to LCL161, TGFβ reduced XIAP levels and slightly increased cIAP1/2 levels in both BV2 and RAW cells (Fig. 6F).

We next aimed to determine the secretion of TGFβ isoforms by murine GBM cells treated with a high dose SMC. All murine GBM cells—CT2A, GL261, and SMA560—secreted TGFβ (TGFβ1 > TGFβ2 > TGFβ3; Fig. 6G). SMA560 expressed significantly higher TGFβ1 relative to CT2A and GL261 cells, in keeping with its canonically high TGFβ expression^77^, which increased further with LCL161 treatment. No significant changes were observed for expression of TGFβ2 or TGFβ3. These data indicate that TGFβ, a key regulator of GBM biology, is secreted by murine GBM cells, is expressed within intracranial GL261 tumors and by surrounding healthy tissue and modulates IAP levels.

Given this, we reasoned that TGFβ blockade would enhance cotherapy, allowing for enhanced TAM clearance, GBM death, and T-cell mediated immunity. The inclusion of α-TGFβ antibodies significantly improved overall survival of mice bearing intracranial GL261 tumors and cotreated with α-PD-1 and LCL161, doubling the number of long-term survivors relative to cotherapy alone (2/8 vs. 5/8; Fig. 6H). This effect was not observed with the lower 75 mg/kg dose. To further characterize immune responses, we analysed the spleens and draining lymph nodes from treated mice. We observed no changes in the total T-cell proportions or CD4^+^ and CD8^+^ subsets (Supplemental Fig. 5A, B) regardless of treatment. However, the addition of LCL161 to any antibody treatment significantly increased the number of CD4^+^, but not CD8^+^, T-cells expressing OX40 and CD25 in lymph nodes (Supplemental Fig. 5C, D, G). TIM3 expression was modestly increased on lymph node CD4^+^ T-cells (Supplemental Fig. 5E, H) and splenic macrophage proportions were reduced relative to α-PD-1 alone (Supplemental Fig. 5F). No changes were observed in the CD11b, Ly6C or Ly6G content (Supplemental Fig. 5I–L). Together, these findings suggest that peripheral immunoactivation is primarily driven by SMC treatment, with PD-1 and TGFβ blockade removing key barriers to effective antitumor immunity.

## Discussion

Despite significant research into the complex biology of GBM, this tumor type remains near uniformly fatal. Treatment resistance arises from multiple factors, including substantial intra- and inter-tumoral heterogeneity, therapy resistant GBM stem cell populations, the immune-restricted CNS, the blood brain barrier limiting drug delivery, and the presence of immunosuppressive cell populations and cytokine signaling networks. We show that murine GBM cells express basal and inducible immune proteins that support recognition and clearance by the adaptive immune system. GL261 cells uniquely induce MHCII expression via IFNγ, consistent with our findings showing anti-GL261 immune memory appears to be CD4^+^ T-cell mediated. Similar to our observations showing that subcutaneous GL261 implants act as de facto vaccinations for subsequent intracranial rechallenge, GBM-specific peptide vaccines have been shown to activate CD4^+^ T-cells, promoting tumor lysis, antigen spreading, and GBM clearance^78^. CD4^+^ T-cells are recognized beyond their conventional T-helper or Treg functions, with tumor-infiltrating CD4^+^ T-cells recognizing MHCII-presented antigens to direct cytotoxic effects in melanoma^79^^,^^80^. In a glioma context, TNF-α and IL-10 upregulate MHCII and LAG3 expression, contributing to CD4^+^ T-cell exhaustion. Blockade of LAG3, TNF-α and IL-10 significantly enhances α-PD-1 efficacy in GL261^81^. In the CT2A model, dysfunctional CD4^+^ T-cells contribute to CD8^+^ T-cell exhaustion and reduced responses to α-PD-1 therapy^82^. GL261 more closely resembles the immune landscape of patient GBM samples compared to CT2A^54^, and its inducible MHCII expression highlights the potential for CD4^+^ T-cell mediated immunity in this model. Nonetheless, GL261 is more inflamed and responsive to α-PD-1 than human GBM, underscoring limitations of current murine GBM models. Thus, the nuanced role of CD4^+^ T-cells in anti-GBM immunity and their interaction with IAP blockade represents a key future area of research.

Most preclinical studies are conducted in young mice, including our prior work on SMC and checkpoint blockade synergy^50^^,^^51^. Both the CNS and immune system undergo substantial age-related changes^83–85^, including increased memory T-cell populations, reduced responsiveness to novel antigens, immunosenescence, increased CNS barrier permeability, and neurodegeneration. It is therefore unsurprising that aged mice showed reduced therapeutic efficacy in both CT2A and GL261 models. Our observation of high IDO1 (an immunosuppressive enzyme) expression in tumor-infiltrating CD45^+^ cells aligns with reports linking high T-cell IDO1 expression to poor survival^86^. Few studies have examined immunotherapy efficacy in aged GBM-bearing mice. Notably, Ladomersky *et al.*reported IDO1 expression and senescent cell populations in aged mouse bearing orthotopic GL261 tumors, with consequent reduced efficacy of radiotherapy, PD-1 blockade, and IDO1 inhibition in 78–86 week (∼20–22 month) old mice^87^, consistent with our findings. We suggest testing anti-GBM immunotherapies in aged animals as a prerequisite to further translational work.

The enhanced efficacy of SMC monotherapy in middle-aged GL261-bearing mice represent an interesting finding requiring further study. SMCs exhibit senolytic activity, promoting apoptosis in treatment-induced senescent GBM cells^88^. cIAP2 is upregulated following temozolomide-induced senescence, and IAP blockade increases cell death of senescent GBM cells^89^. These senolytic and immunoactivating effects may explain the improved survival with LCL161 monotherapy in middle-aged animals, particularly in the GL261 model. Confirming these effects *in vivo* is a promising future area of research, as will combination with IDO1 inhibitors, which have shown promising preclinical^90–92^ and early clinical trial results^93^^,^^94^. These agents beneficially reshape the tumor microenvironment when combined with other agents including α-PD-1 checkpoint blockade and the GBM standard of care Stupp protocol (radiotherapy plus temozolomide)^95^^,^^96^.

We show that increasing the dose of SMCs reduces TAM populations and increases the proportion of microglia within the tumor microenvironment. These *in vivo* TAM reducing effects by SMCs are consistent with our previous findings *in vitro*^50^. The lack of significant differences in CC3 expression potentially reflects issues with timing of analysis relative to the last LCL161 treatment, as our *in vitro* studies showed rapid caspase-3 activation within a few hours of LCL161 treatment and cell death of murine macrophages^50^. It is therefore feasible we analyzed tumors at a time where the affected macrophages had undergone apoptosis and were phagocytosed and replaced by first responder microglia. Future work will look to examine the dynamics of anti-GBM immunity comparing populations at different times post-implant and post-SMC treatment. A higher microglia-to-macrophage ratio is associated with better prognoses and more pro-inflammatory gene signatures^97^, which may explain the significant survival benefit observed with an increased SMC dosing. However, high frequency SMC treatment combined with checkpoint blockade resulted in significantly worse survival outcomes despite near complete tumor eradication in the treated cohort. This paradoxical effect was accompanied by depletion of SSC^low^ FSC^mid^ populations in lymph nodes and spleens by 5×/wk high dose SMC treatment, matching similar reductions in splenic T-cells following intracranial GL261 implantation. These findings suggest that this regimen induces severe lymphodepletion.

Consistent with our findings of enhanced T-cell costimulation following SMC treatment, engineered T-cells exhibit enhanced costimulatory signaling but increased sensitivity to Fas-mediated apoptosis following IAP blockade^98^. In a GBM context, astrocyte-expressed FasL can induce apoptosis of brain-infiltrating T-cells^99^. Given the increased astrocyte involvement around GBM tumors following SMC treatment^50^, SMCs may contribute to T-cell death in this context. These effects may explain the lack of significant change in intratumoral T-cell populations despite significant peripheral immunoactivation. Future work will focus on developing strategies for enhanced intratumoral SMC delivery to maximize TAM and GBM killing. Along these lines, intratumoral delivery of temozolomide and other chemotherapeutics has been shown to elicit stronger anti-GBM immune responses and survival outcomes in combination with α-PD-1, compared to systemic delivery. Systemic administration was associated with significant immune defects^100^^,^^101^ similar to our high-dose/high-frequency SMC treatment. Importantly, twice weekly high dose SMC treatment significantly increased peripheral immunoactivation, laying the foundation for enhanced therapeutic efficacy when combined with agents removing key immunosuppressive hurdles, such as PD-1 and TGFβ blockade. Within our IHC analysis, the only significant T-cell mediated change observed following SMC treatment was an increase in TIM3 among the CD8^+^ fraction, suggesting this immune checkpoint may also be a rational target for improving SMC-mediated therapies. TIM3 has been shown to play key roles in GBM immunosuppression^102^^,^^103^ but single agent TIM3 blockade has shown no significant preclinical survival benefit in the GL261 model^104^^,^^105^, further supporting combination with SMCs and PD-1 blockade.

The pleiotropic cytokine TGFβ has a wide range of context-dependent effects, including immunosuppression. TGFβ blockade has the potential to enhance immunotherapies for a wide array of cancers^106–108^. Inhibitors targeting TGFβ and/or its upstream regulators are currently in clinical trials for various solid tumors, including in combination with α-PD-1 or α-CTLA4, with encouraging early results^109–111^. TGFβ is highly expressed in GBM and plays critical roles in tumor aggressiveness and immunosuppression^67^. Several clinical trials targeting TGFβ2, TGFβR1, or pan-TGFβ signaling in GBM have been completed or are underway^112^, and shown to be safe but with minimal clinical benefit^113–115^. In GL261 models, TGFβ blockade combined with GBM antigen vaccination enhanced anti-tumor immunity and survival^17^. Similar survival improvements in GL261-bearing animals were seen when TGFβ blockade was combined with radiotherapy and α-PD-L1 treatment^116^^,^^117^. We have previously shown that TGFβ protects microglia and macrophages from SMC-mediated cytotoxicity^50^ and here we show that *in vivo* TGFβ blockade in the context of SMC and α-PD-1 cotherapy nearly triples the number of long-term survivors in GBM-bearing mice relative to isotype control. This improvement was only noted in the high dose SMC group. Within these combination therapies, SMCs function as the primary peripheral immunostimulant while TGFβ and PD-1 blockade remove immunosuppressive and anti-apoptotic barriers, enabling maximal anti-GBM efficacy. Future work will focus on optimizing the timing and dosing of TGFβ blockade to enhance TAM killing and GBM clearance, alongside strategies to improve SMC delivery to the tumor site. Together, these findings highlight that maximizing intratumoral SMC exposure and incorporating TGFβ blockade are promising therapeutic avenues for improving outcomes in this highly lethal cancer.

## Supplementary Material

vdaf253_Supplementary_Data

## Data Availability

The datasets generated during and/or analysed during the current study are available upon on reasonable request.
