# Supplementary figures and images for: Enhanced glioblastoma immunotherapy via SMAC mimetic dose escalation and TGFβ blockade
Source: Neurooncol Adv. 2025 Dec 10;8(1):vdaf253. doi: 10.1093/noajnl/vdaf253 (PMC12901662; doi:10.1093/noajnl/vdaf253)

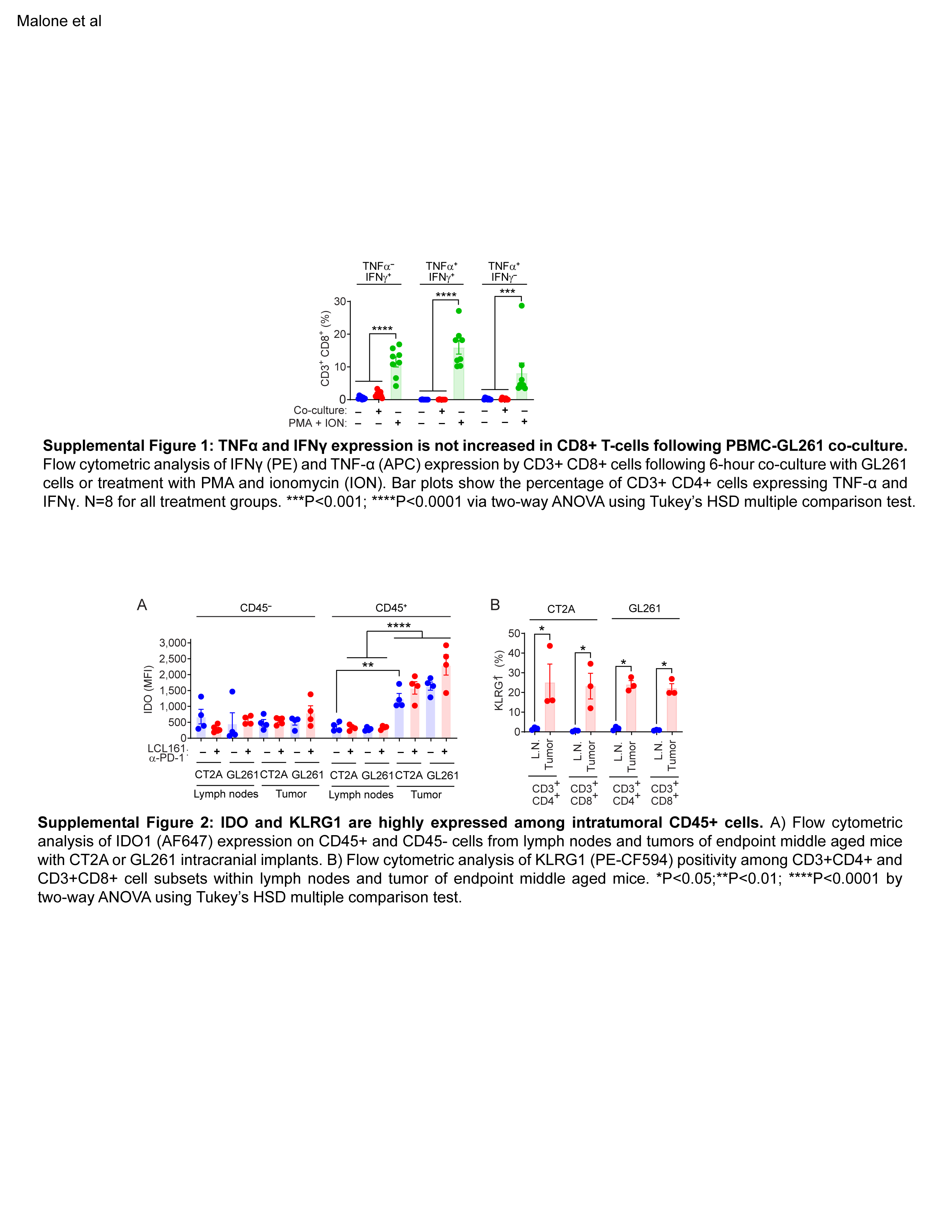

Supplement: vdaf253_Supplementary_Data [file vdaf253_supplementary_data.zip › Paper 2 figures v7.5 individual - Supp figures 1,2.tif]

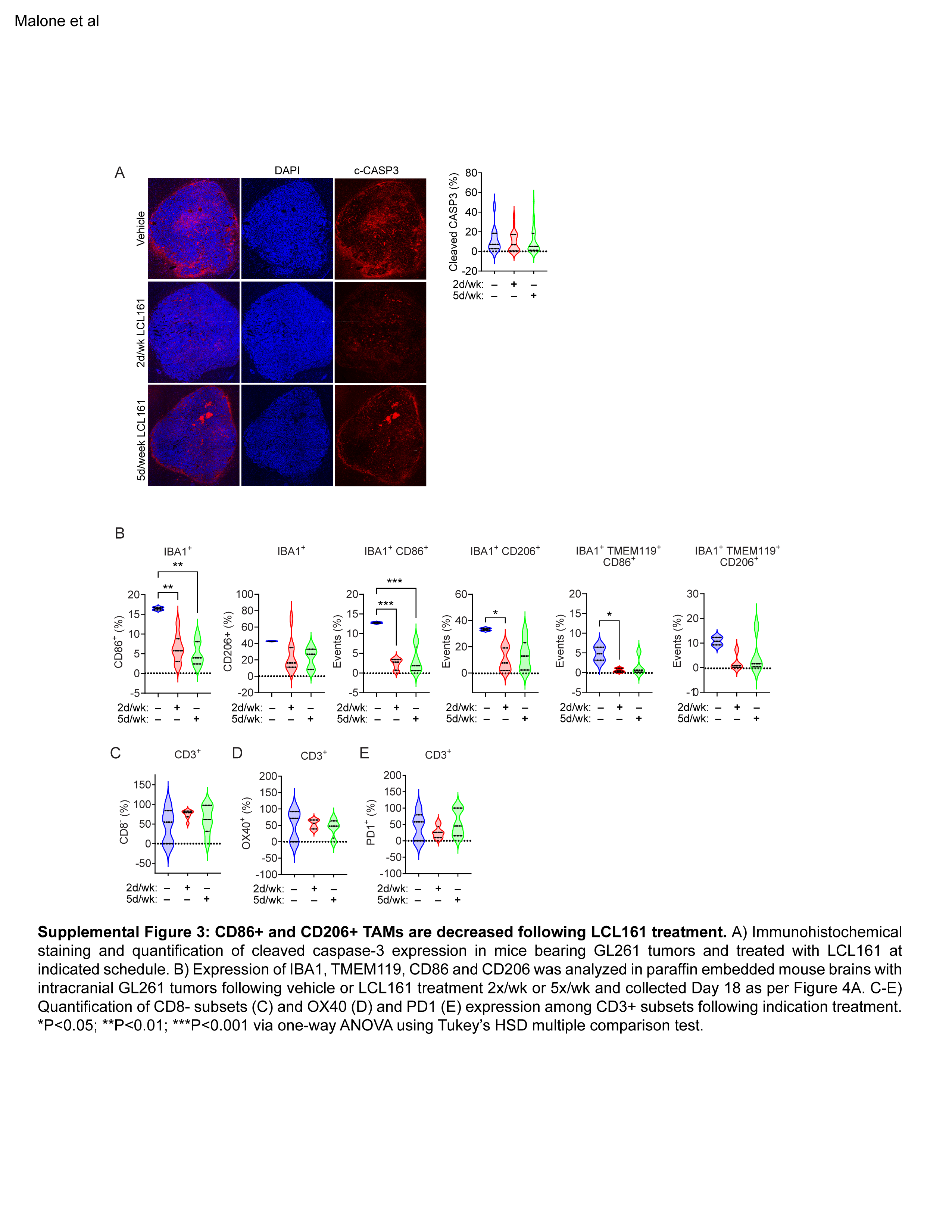

Supplement: vdaf253_Supplementary_Data [file vdaf253_supplementary_data.zip › Paper 2 figures v7.5 individual - Supp figures 3.tif]

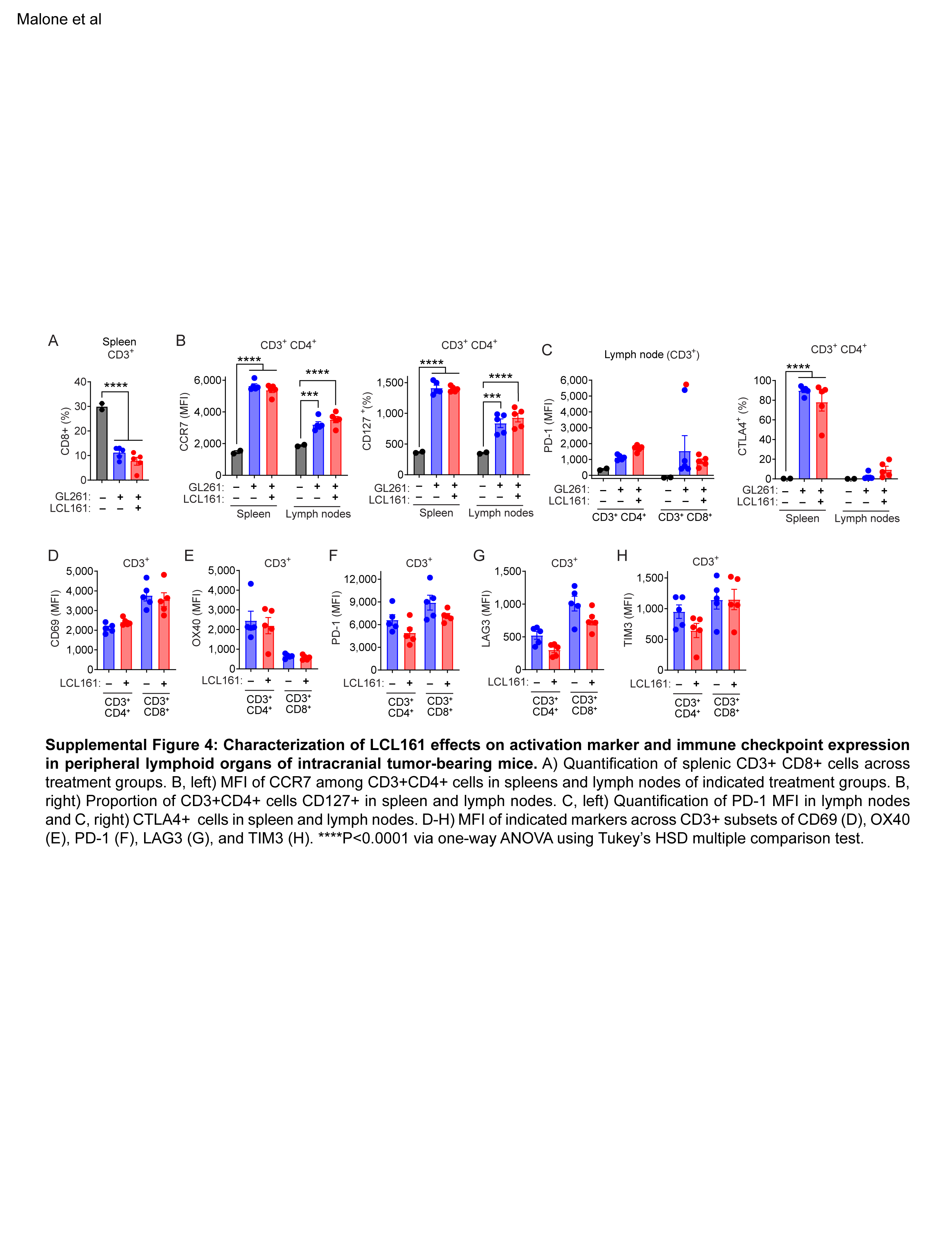

Supplement: vdaf253_Supplementary_Data [file vdaf253_supplementary_data.zip › Paper 2 figures v7.5 individual - Supp figures 4.tif]

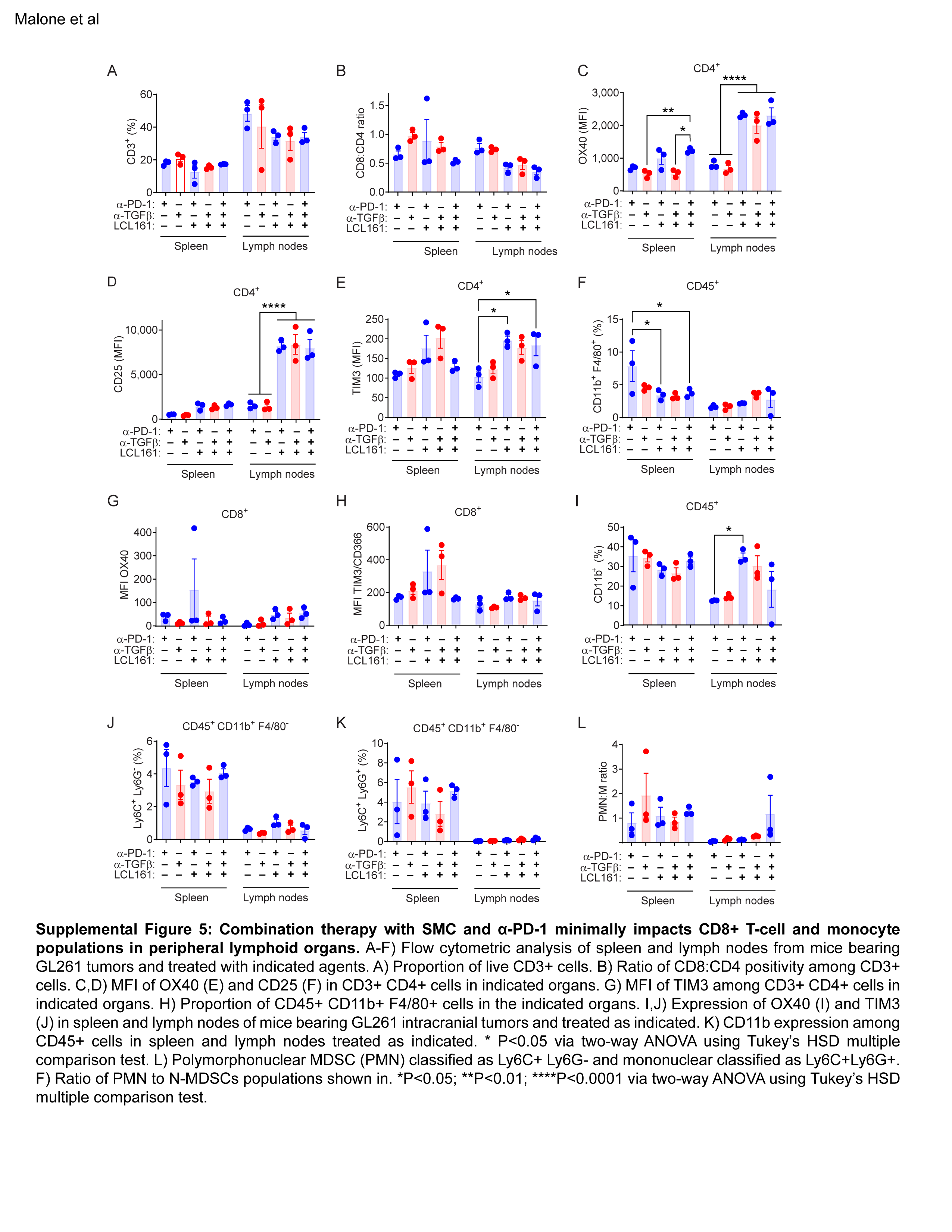

Supplement: vdaf253_Supplementary_Data [file vdaf253_supplementary_data.zip › Paper 2 figures v7.5 individual - Supp figures 5.tif]

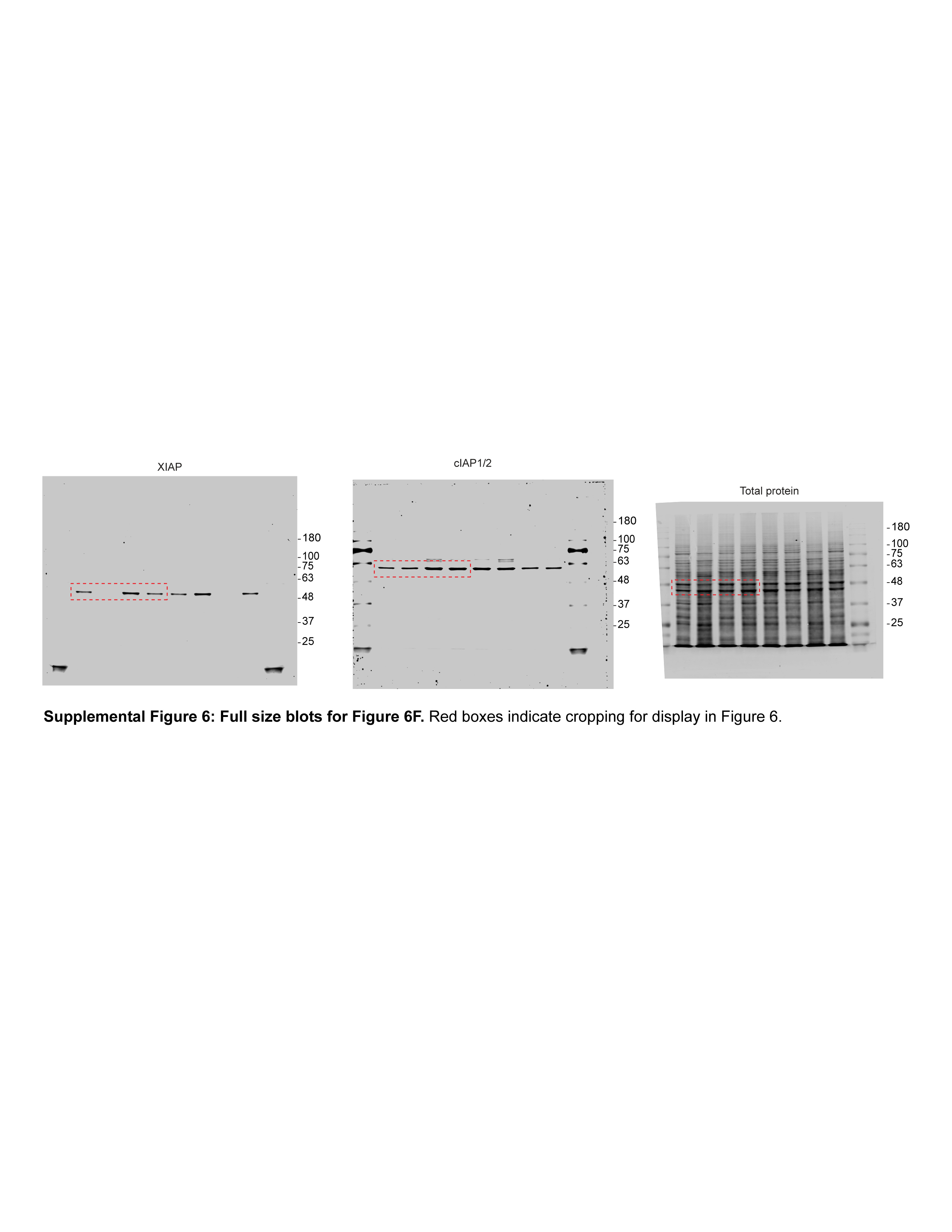

Supplement: vdaf253_Supplementary_Data [file vdaf253_supplementary_data.zip › Paper 2 figures v7.5 individual - Supp figures 6.tif]
